# Correction: CRISPR/Cas9-mediated generation of biallelic F0 anemonefish (*Amphiprion ocellaris*) mutants

**DOI:** 10.1371/journal.pone.0305644

**Published:** 2024-06-12

**Authors:** Laurie J. Mitchell, Valerio Tettamanti, Justin S. Rhodes, N. Justin Marshall, Karen L. Cheney, Fabio Cortesi

In Fig 4, there is an error in panel C. The label of x-axis for the bar plot of Fig 4C should have been M5, M6, M7. Please see the correct Fig 4 here.

**Fig 4:**
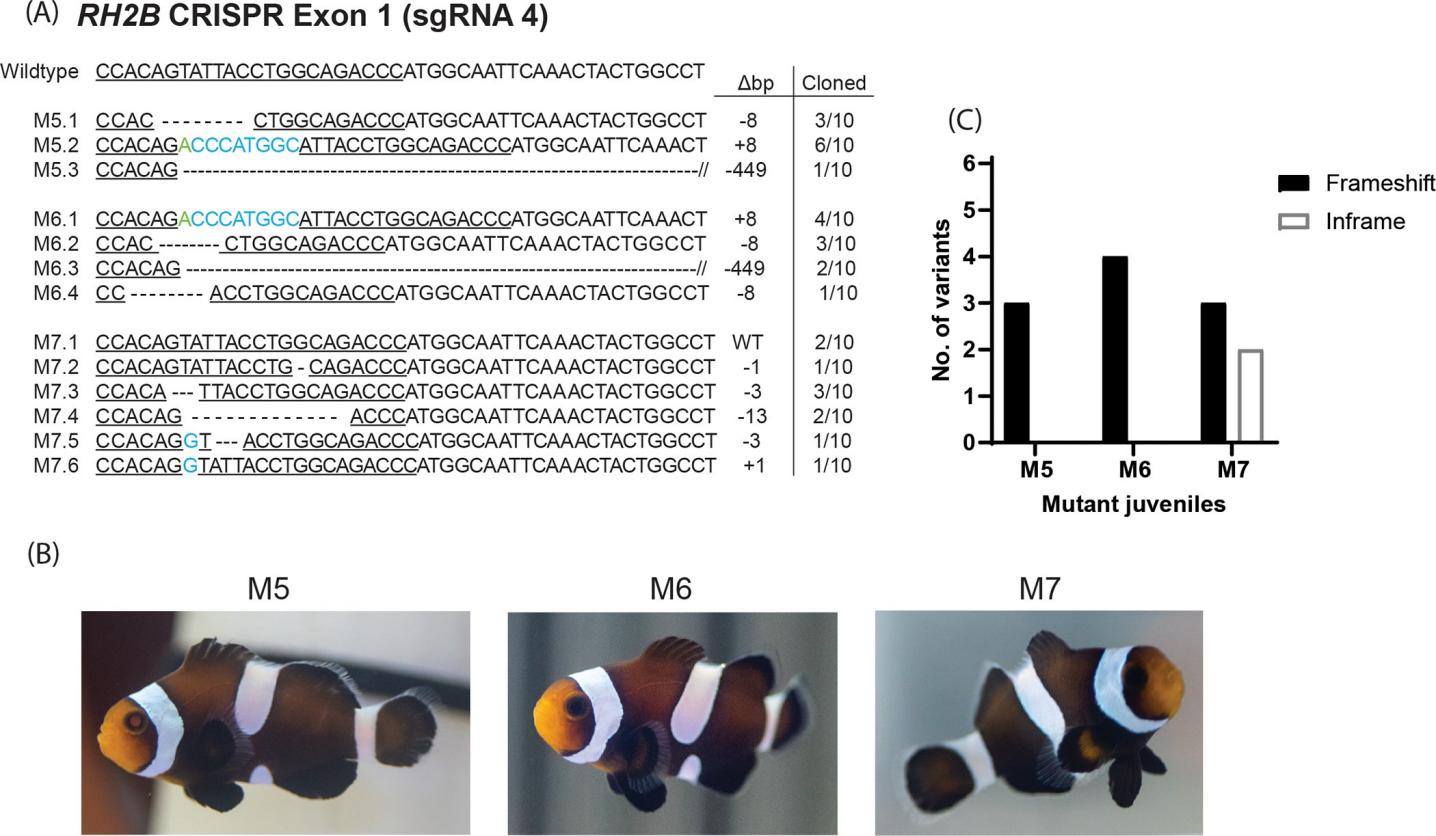
Genotype analysis of four-month-old RH2B mutant *Amphiprion ocellaris*. (A) Subcloned sequences belonging to A. ocellaris juveniles (clutch 11, sgRNA RH2B 4) with mutations at targeted sequences (underlined) located on Exon 1 of the RH2B opsin gene. Wildtype (WT) sequence is included for reference. Mutations included deletions (dashes), substitutions (green), and insertions (blue). Sequence labels on the left-side indicate mutant fish and allele no., while numbers on the right-side indicate the base pair change (Δbp) and the proportion of each allele out of the total number of cloned sequences for each fish. (B) Images of the RH2B mutant A. ocellaris juveniles. (C) Number of frameshift and in-frame mutations per RH2B mutant fish.
