# Supplementary material for: CRISPR/Cas9-mediated generation of biallelic F0 anemonefish (Amphiprion ocellaris) mutants
Source: PLoS One. 2021 Dec 15;16(12):e0261331. doi: 10.1371/journal.pone.0261331 (PMC8673619; doi:10.1371/journal.pone.0261331)
Supplement: S1 File — List of injected sgRNA sequences. (DOCX) [file pone.0261331.s001.docx]

**S1 sgRNA sequences**

*RH2B* sgRNA

Target sequence 1: *RH2B* Exon 4 (‘5 – ‘3) GGCCAUGGAGACAGCUGAGA

Target sequence 2: *RH2B* Exon 5 (‘5 – ‘3) UGGAAUGGGUGGCAUGGUUG

Target sequence 3: *RH2B* Exon 5 (‘5 – ‘3) CUGAGCACCAUUGGAAUGGG

Target sequence 4: *RH2B* Exon 1 (‘5 – ‘3) GGGUCUGCCAGGUAAUACUG

*TYR* sgRNA

Target sequence 1: *TYR* Exon 2 (‘5 – ‘3) GGCGUUGUGUAACGCAACA

Target sequence 2: *TYR* Exon 2 (‘5 – ‘3) GAGGAGUACAACAGCCGAG
