# Supplementary material for: CRISPR/Cas9-mediated generation of biallelic F0 anemonefish (Amphiprion ocellaris) mutants
Source: PLoS One. 2021 Dec 15;16(12):e0261331. doi: 10.1371/journal.pone.0261331 (PMC8673619; doi:10.1371/journal.pone.0261331)
Supplement: S2 File — Primer sequences and PCR routine, and in-vitro cutting assay reagents and incubation steps. (DOCX) [file pone.0261331.s002.docx]

**S2 sgRNA *in-vitro* assay**

For further details on the *in-vitro* assay (e.g. reaction buffer ingredients) see the original protocol outlined by Grainger *et al.* 2017.

Reaction mix:

PCR product 8 μL

10X Cas9 reaction buffer 2 μL

Cas9 protein 2 μL

sgRNA 1 μg

Nuclease free water to 20 μL

Incubation steps:

1. Incubate the above mix at 37°C for 1 h, and 98°C for 20 min to denature excess Cas9 protein. Store at -4°C.
2. Run on a 2.5-3.0% agarose gel to analyse cleavage activity Cas9 directed by your sgRNA by comparison with PCR product without Cas9 or sgRNA.

**PCR primer sequences**

RH2B

Forward primer (‘5 – ‘3) TCCCAGTACACAACGCAGTC

Reverse primer (‘5 – ‘3) AGGGTGTCTGGAGATGTGGA

TYR

Forward primer (‘5 – ‘3) ACCAGTCGACACTTGCTGCT

Reverse primer (‘5 – ‘3) TCGGTCCATAGGTCCCGTCT

**PCR routine**

See New England Biolabs (product code: M0496S) for recommended reagent quantities for a desired reaction volume.

Thermocycler steps:

Initial denaturation 95°C (for 30 seconds)

Repeated for 30 cycles 95°C (15 seconds)

60°C (60 seconds)

68°C (30 seconds)

Final extension 68°C (5 minutes for in-vitro test; 10 minutes for subcloning)

Hold 4°C (forever)
