# Supplementary material for: CRISPR/Cas9-mediated generation of biallelic F0 anemonefish (Amphiprion ocellaris) mutants
Source: PLoS One. 2021 Dec 15;16(12):e0261331. doi: 10.1371/journal.pone.0261331 (PMC8673619; doi:10.1371/journal.pone.0261331)
Supplement: S3 File — List of reagents and quantities used to make a salt-balanced solution for eggs. (DOCX) [file pone.0261331.s003.docx]

**S3 Yamamoto’s ringer’s solution:**

0.75% NaCl

0.02% KCl

0.02% CaCl2

0.002% NaHCO3

(Adjusted to pH 7.3 with NaHCO3)

Yamamoto’s ringer’s solution references:

Yamamoto, T. (1939). Changes of the cortical layer of the egg of *Oryzias latipes* at the time of fertilisation. *Proceedings of the Imperial Academy (Toykyo)*, *15*, 269-271.

Kinoshita, M., Murata, K., Naruse, K., & Tanaka, M. (2009). Appendix 3 Solutions. In *Medaka biology, management, and experimental protocols* (pp. 397)*.* Wiley-Blackwell, Singapore.
