# Supplementary material for: CRISPR/Cas9-mediated generation of biallelic F0 anemonefish (Amphiprion ocellaris) mutants
Source: PLoS One. 2021 Dec 15;16(12):e0261331. doi: 10.1371/journal.pone.0261331 (PMC8673619; doi:10.1371/journal.pone.0261331)
Supplement: S4 File — Microcapillary settings and pneumatic microinjector settings. (DOCX) [file pone.0261331.s004.docx]

**S4 Crafting of microneedles**

Microneedles for CRISPR-construct delivery were pulled from borosilicate capillaries (Harvard Apparatus: 1.0x0.58x100 mm) using a micropipette puller (P-1000 Sutter Instruments with a 2 mm wide trough filament) to produce microneedles with a short (3-4 mm) taper. This was found to be important in minimising the bending of the needle tip when attempting to penetrate the thick chorion of anemonefish eggs. Our used micropipette puller settings were as follows,

Heat: +10% Ramp value Pull: 55 Vel.: 70 Time: 165 Pressure: 500

Microneedles were viewed under a microscope against a 0.01mm scale calibration slide, and tips were broken back using a pair of fine-tipped forceps to give a final tip diameter between 10-15 μm.

**Calibration of microinjector settings**

Pre-cut needles were backloaded with 2 μL of phenol red dye mixed with water and fastened on the microinjector (Narishige IM-400). Air pressure and pulse duration settings were adjusted to produce a single droplet diameter of 125-140 μm measured on a calibration slide (0.01 mm scale) that roughly corresponded to a volume between 1-1.4 nL. Our used injector settings were as follows,

Pressure: 1.017 psi Time: 0.8-1.0 sec. Back pressure: 0 psi (on)
