# Supplementary figures and images for: CRISPR/Cas9-mediated generation of biallelic F0 anemonefish (Amphiprion ocellaris) mutants
Source: PLoS One. 2021 Dec 15;16(12):e0261331. doi: 10.1371/journal.pone.0261331 (PMC8673619; doi:10.1371/journal.pone.0261331)

**
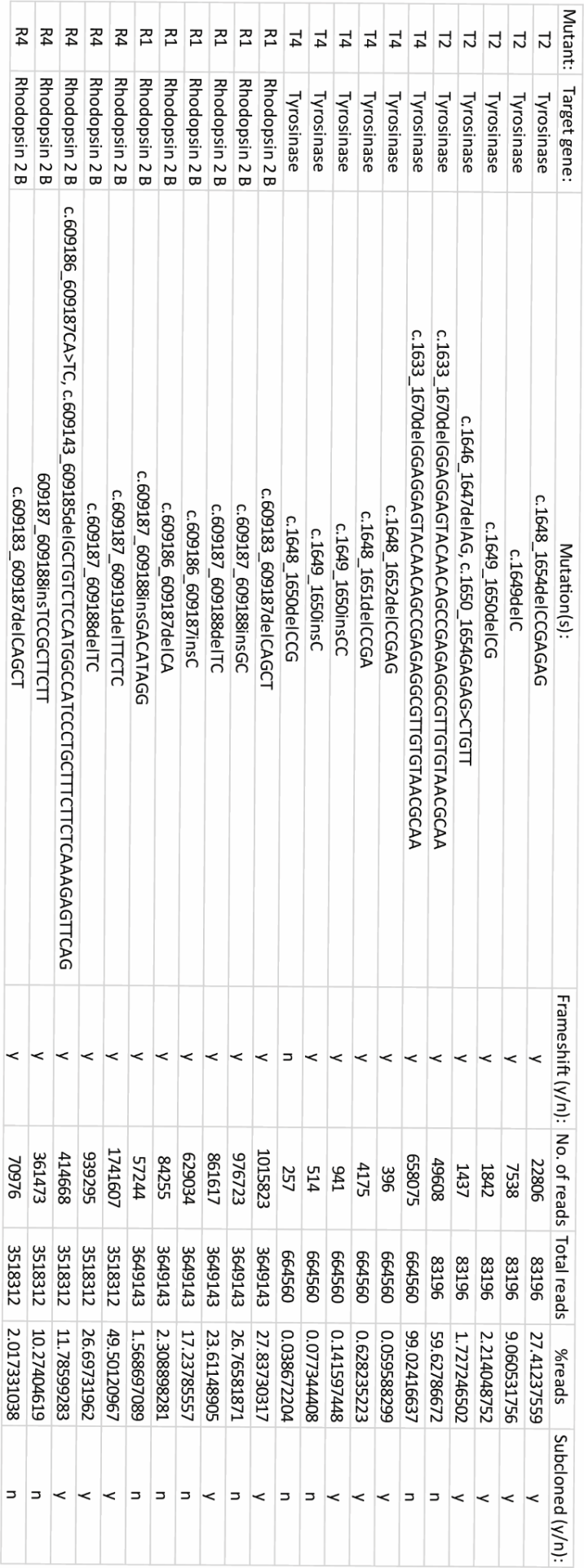
S5 Full summary of NGS variant sequences**

Supplement: S5 File — (DOCX) [file pone.0261331.s005.docx]

RH2B in-vitro assay

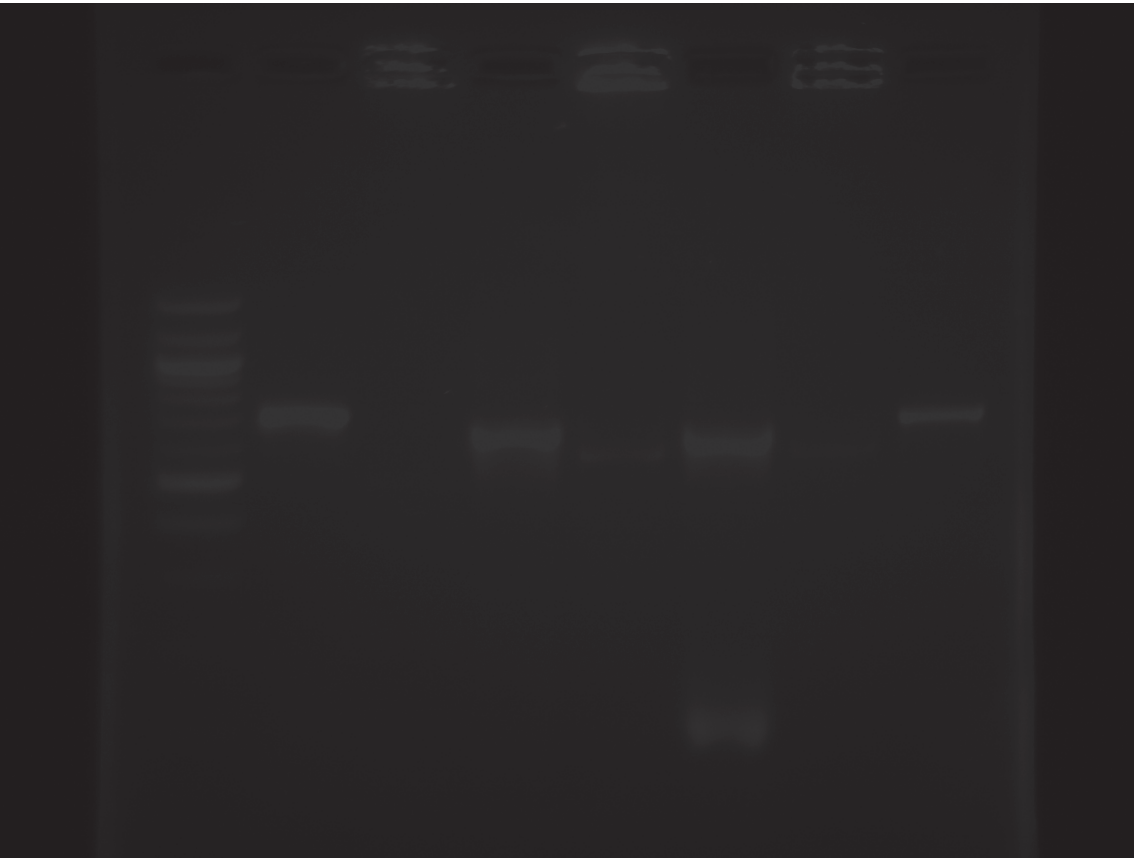

tyr in-vitro assay

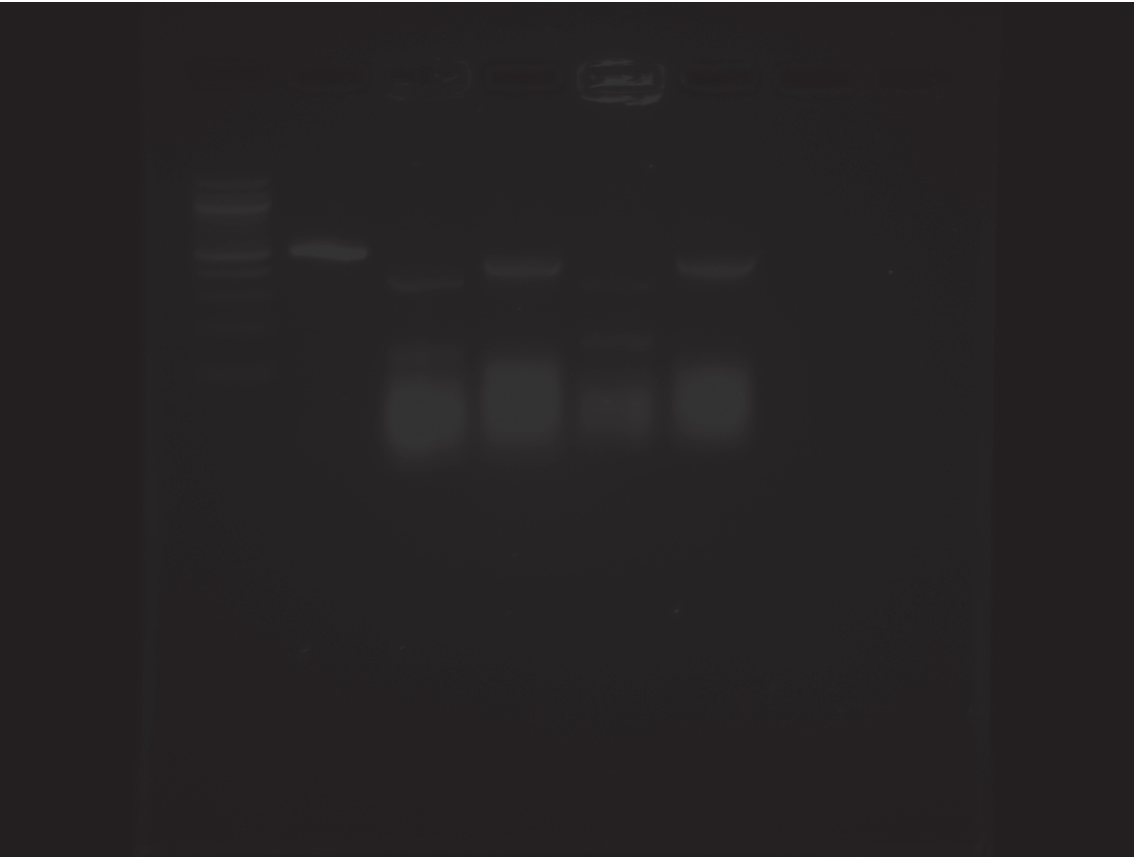

Supplement: S1 Raw images — (PDF) [file pone.0261331.s007.pdf]
