# Supplementary material for: CRISPR/Cas9-mediated generation of biallelic F0 anemonefish (Amphiprion ocellaris) mutants
Source: PLoS One. 2021 Dec 15;16(12):e0261331. doi: 10.1371/journal.pone.0261331 (PMC8673619; doi:10.1371/journal.pone.0261331)
Supplement: S6 File — Translated sequence alignment of frameshifted alleles found in RH2B-M1 and -M4, and wildtype (WT) RH2B for reference. Sequences were aligned against bovine rhodopsin (RH1) (NCBI accession no. NP_001014890.1), as an opsin template. The chromophore binding site (bovine RH1 AA no., Lys296) is boxed in blue. Amino acid (AA) numbering schemes were according to WT RH2B (upper) and bovine RH1 (lower). Translated sequences were aligned using MAFFT Alignment (v7.450) in Geneious. MAFFT reference: Katoh, K., & Standley, D. M. (2013). MAFFT Multiple Sequence Alignment Software Version 7: Improvements in Performance and Usability. Molecular Biology and Evolution, 30(4), 772–780. https://doi.org/10.1093/molbev/mst010. (DOCX) [file pone.0261331.s006.docx]

**S6 Translated *RH2B* sequence alignment**

**
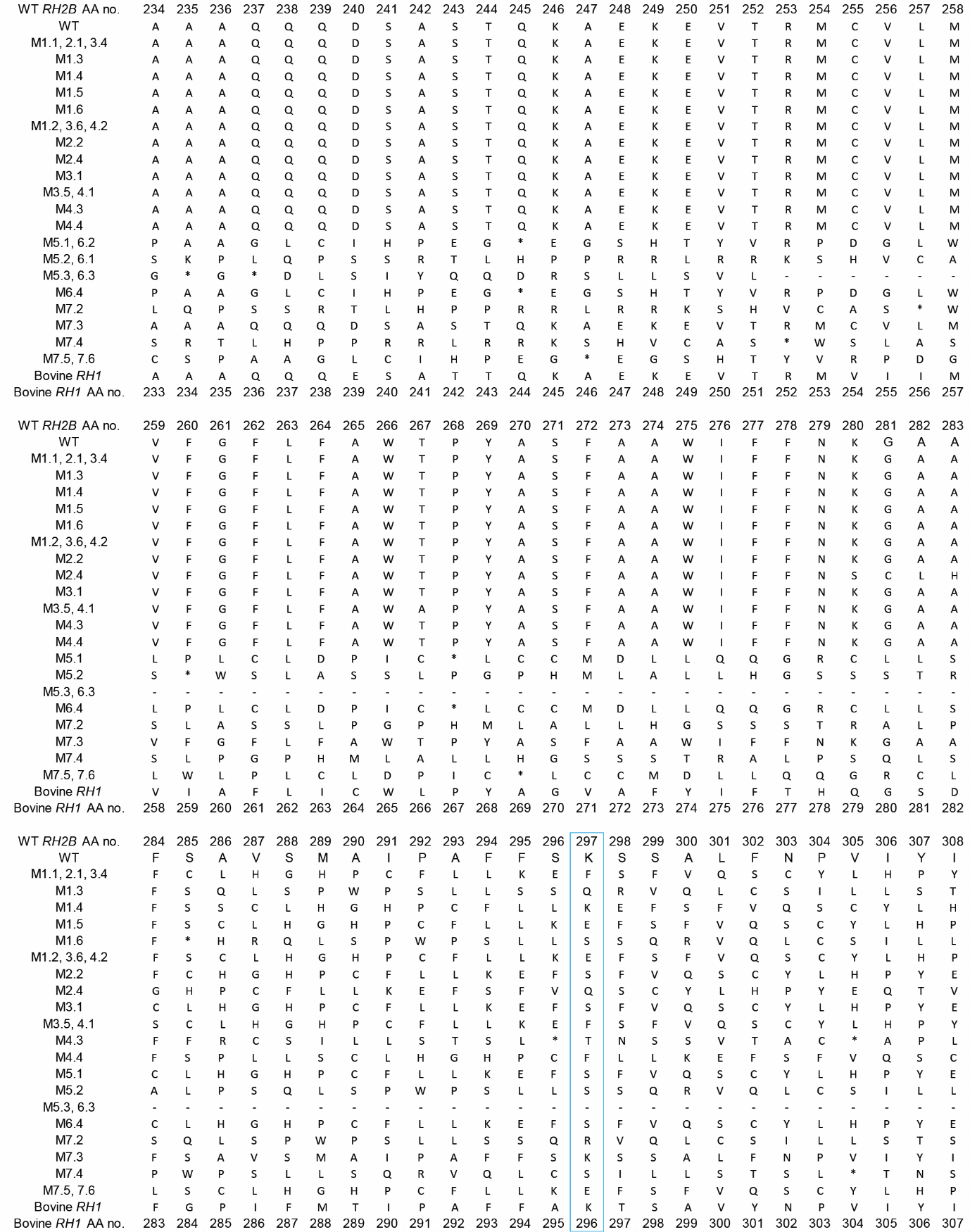
**

Translated sequence alignment of frameshifted alleles found in *RH2B*-M1 and -M4, and wildtype (WT) *RH2B* for reference. Sequences were aligned against bovine rhodopsin (*RH1*) (NCBI accessio n no. NP_001014890.1), as an opsin template. The chromophore binding site (bovine *RH1* AA no., Lys296) is boxed in blue. Amino acid (AA) numbering schemes were according to WT *RH2B* (upper) and bovine *RH1* (lower). Translated sequences were aligned using MAFFT Alignment (*v7.450*) in Geneious.

MAFFT reference: Katoh, K., & Standley, D. M. (2013). MAFFT Multiple Sequence Alignment Software Version 7: Improvements in Performance and Usability. *Molecular Biology and Evolution*, *30*(4), 772–780. https://doi.org/10.1093/molbev/mst010
